# Chest wall reconstruction in benign and malignant tumors with non-rigid materials: An overview

**DOI:** 10.3389/fsurg.2022.976463

**Published:** 2022-08-03

**Authors:** Sara Colella, Alessandro Brandimarte, Roberta Marra, Stefano Marinari, Armida D’Incecco, Milena Di Genesio Pagliuca, Andrea De Vico, Roberto Crisci, Duilio Divisi

**Affiliations:** ^1^ Unit of Respiratory Diseases, “G. Mazzini” Hospital, Teramo, Italy; ^2^ Unit of Respiratory Diseases, “V. Fazzi” Hospital, Lecce, Italy; ^3^ Medical Oncology Unit, “Giuseppe Mazzini” Hospital, Teramo, Italy; ^4^ Radiotherapy Unit, “Giuseppe Mazzini” Hospital, Teramo, Italy; ^5^ Department of Life, Health and Environmental Sciences, University of L’Aquila, Thoracic Surgery Unit, “Giuseppe Mazzini” Hospital, Teramo, Italy

**Keywords:** chest wall tumors, resection, reconstruction, mesh, myoplastic

## Abstract

Several materials and techniques have been described for the procedure of chest wall reconstruction: the choice of using a technique or a material over another relies mainly on the surgeon's experience as well as thoracic defect localization and dimension, local availability of materials, and costs. From a technical point of view, autologous and alloplastic reconstruction are available, and, in both cases, rigid and non-rigid prostheses are found. Each material has its peculiarities, with advantages and disadvantages; thus, it is mandatory to be confident when planning the intervention to foresee possible complications and minimize them. We have reviewed the literature on chest wall reconstruction in chest wall tumors (both malignant and non malignant) with non-rigid prosthetic materials, focusing on safety outcomes.

## Introduction

Reconstruction of the thoracic wall is indicated in case of cancers (either primary or metastatic), traumas, infections, and congenital defects: these conditions could result in functional and aesthetic impairments of the thoracic wall; thus, the aim of surgical operation is to protect intrathoracic structures, to preserve cardiac and respiratory functions, to avoid upper extremity instability, to receive radiotherapy when indicated, and to assure the best aesthetic result possible. In addition, in the case of cancer, the aim of surgery is to obtain the wider margins possible, free from malignant disease, or for palliative purposes (1, 2).

No strict indications are available about the size and the location of defects that need to be reconstructed nor about the material that has to be used; the reconstruction strategy is mainly up to the surgeon’s experience, to the local availability of materials, and to cost-effective analysis. Usually, defects smaller than 5 cm in any part of the thorax tend not to be reconstructed, as well as defects smaller than 10 cm and located in the posterior part of the thorax, because the protection and support given by the scapula do not alter thoracic and arm movements (3). All other chest wall defects need to be evaluated for surgical reconstruction, and part of the intervention plan consists in choosing the most suitable material for the chest wall reconstruction.

Methods for reconstruction of the thoracic wall could be categorized mainly into two groups: rigid and non-rigid prostheses. An ideal prosthetic material should be rigid enough to avoid chest paradoxical respiratory movements, should be inert and let the growth of fibrous tissue without the risk of infection, should be easily shaped during the operation, should be radiolucent to enable an anatomic reference for relapses detection during the follow-up, and should be not expensive (4).

The aim of this analysis is to review surgical outcomes of chest wall reconstruction, focusing on non-rigid materials in chest wall tumors in terms of mortality, quality of life, complications, and length of hospital stay.

## Methods

A literature search was performed on PubMed using keywords “chest wall,” “reconstruction,” “semi-rigid,” “non-rigid,” “tumors,” “cancer,” “malignant,” “non malignant,” “benign,” and “diseases,” excluding case reports and studies with less than 10 patients.

Only articles in English were included and published from 2000 to this day.

Studies evaluating congenital defects, infections, and trauma as indications for chest wall reconstruction were excluded.

For each study, the following data were collected: type of the study, number of patients included, materials used for the reconstruction of the thoracic wall, if sternectomy was performed, 30-day mortality, need for reintervention, length of hospital stay, quality of life, and complications such as local infection, hematoma, fistula, pneumonia, necrosis, dehiscence of the surgical wound, paradoxical respiratory movement, and flap lose.

## Results

After the first search, 1,343 records were found, and a second selection was made by the title, extracting 102 papers. Of these 102 papers, 16 were finally chosen for review (5–20). Studies evaluating the combination of rigid and semi-rigid prostheses were included only if a subgroup analysis for semi-rigid materials was done.

All but one were retrospective studies—one prospective noncomparative—; of these, two studies compared the outcomes of non-rigid prostheses with rigid ones retrospectively. The total amount of patients in the 16 studies was 1,089, ranging from 11 to 345. Indications for chest wall reconstruction were benign tumors (desmoid tumor, angiomyolipoma, and neurofibromas) and malignant tumors (sarcoma, lung cancer, metastases, breast cancer, melanoma, mediastinal tumors, and bone and cartilage tumors).

Materials used for the reconstruction of the thoracic wall were synthetic meshes (Gore-Tex, Vicryl, polypropylene, polytetrafluoroethylene, polyurethane, polyester, polyglycolic acid) in 16 studies and biologic meshes in 6 (acellular collagen matrix, homologous dura mater, swine dermal collagen matrix), associated or not with flaps. In 197 cases, patients also received a partial or total sternotomy.

Thirty-day mortality was reported in every study, ranging from 0% to 5.4% (average: 1.04%, 6 deaths/1,089).

A reintervention was necessary in 34 cases in the overall population due to complications related to the first operation, and in the study of Matrai et al. (12), 14 patients were reoperated because of recurrent disease.

Regarding complications, local infection was investigated in 11 studies, ranging from 2% to 17.3% (42 patients/1,089); hematoma was found in 6 studies, ranging from 2% to 9.1% (15 patients/1,089); fistula was reported only in the study of Daigeler et al. (7), being found in 7 out of 92 (7.6%) patients. Paradoxical movements of the chest wall were reported in 5 cases: in the studies by Daigeler et al. (7) and Huang et al. (19), 25 out of 92 (69.4%) cases and 1 out of 37 (2%) cases were reported; in the other 3 studies, the percentage of these complications was 0%. Pneumonia was considered in 10 studies, ranging from 1.08% to 11.3% (53 patients/1,089); necrosis in 6 studies, ranging from 1.9% to 22% (29 patients/1,089); dehiscence in 5 studies, ranging from 0% to 27% (31 patients/1,089); respiratory failure in 4 studies, ranging from 2.3% to 5.4% (15 patients/1,089); and flap lose in 3 studies, ranging from 2% to 27% (10 patients/1,089).

The only study investigating the quality of life and patients' satisfaction was of Daigeler et al. (7): the aesthetic appearance was considered sufficient or more by the majority of patients, whereas, compared to before the operation, the quality of life was described much better in the 24% of cases, slightly better in the 14%, unchanged in the 19%, slightly worse in the 35%, and much worse in the 8%.

Six studies reported the length of hospital stay, ranging from 1 to 130 days in the ward and from 1 to 74 days in the intensive care unit.

Tables 1 and 2 provide a detailed description of each study.

**Table 1:** Characteristics and data of the included studies (I).

Study number	Author	Year	Type of study	No. of patients	Indication	Partial or total sternectomy no.	Material used
1	Bosc R (5)	2011	Retrospective	22	Breast cancer, melanoma, sarcoma, invasive skin	10	Flap, Gore-Tex, Vicryl
2	D'Amico G (6)	2008	Retrospective	11	Sarcoma	0	Non-cross-linked swine dermal collagen matrix mesh
3	Daigeler A (7)	2009	Retrospective	92	Sarcoma, breast cancer, melanoma, adenocarcinoma, desmoid tumor, angiomyolipoma	35	Prolene, flap
4	Friesenbichler J (8)	2014	Retrospective	31	Primary malignant bone tumor, soft tissue sarcoma	6	Prolene, Vicryl, Gore-Tex, Parietene, Surgisis, Parietex
5	Hanna W (9)	2011	Retrospective	37	Sarcoma, metastases, desmoid, neurofibromas	25	Polypropylene, polyester, polytetrafluoroethylene, polyglactine, autologous
6	Hayashi T (10)	2019	Retrospective	68	Lung cancer, bone and soft tissue tumor	1	Composix, Gore-Tex
7	Leuzzi G (11)	2015	Retrospective	175	Primary chest wall tumor, metastases, benign tumors	35	Vicryl, Gore-Tex, flap
8	Matrai Z (12)	2011	Retrospective	28	Desmoid tumor	NR	Vicryl, Gore-Tex, prolene
9	Spicer J (13)	2016	Retrospective (comparative with rigid prostheses)	345	Primary chest wall tumor, lung cancer, metastases, pleural malignanancy, mediastinal tumors, others	24	Marlex, prolene, Gore-Tex, Dexon, Surgimend, Strattice
10	Tsukushi S (14)	2015	Retrospective	50	Primary chest wall tumor, mestastases	4	Marlex, Gore-Tex, flap
11	van Geel A (15)	2010	Retrospective	60	Soft tissue cancer	13	Homologous dura mater, polyurethane, Vicryl, polytetrafluoroethylene, flap
12	Wald O (16)	2020	Prospective	25	Bone and cartilage tumor, soft tissue cancer	2	Polytetrafluoroethylene, flap
13	Girotti P (17)	2011	Retrospective (comparative with rigid prostheses)	52	Primary chest wall tumor, metastases, benign tumors	52	Soft mesh, flap
14	Schoeder-Finckh A (18)	2019	Retrospective	45	Primary chest wall tumor, metastases, lung cancer	11	Polypropylene, flap
15	Huang H (19)	2015	Retrospective	37	Primary chest wall tumor, metastases, benign tumor	1	Goretex, Marlex, flap
16	Akiba (20)	2012	Retrospective	11	Lung cancer, breast cancer, chondrosarcoma, metastases	2	Gore-Tex, dual mesh, bard composite

**Table 2:** Characteristics and data of the included studies (II).

Study number	30-day mortality, % (no.)	Reintervention, % (no.)	Local infection, % (no.)	Hematoma, % (no.)	Fistula, % (no.)	Pneumonia, % (no.)	Necrosis, % (no.)	Dehiscence, % (no.)	Paradoxical movement, % (no.)	Respiratory failure, % (no.)	Flap lose % (no.)	LOS, days	Length ICU, days
1	4.5% (*n* = 1)	13% (*n* = 3)	13% (*n* = 3)	NR	NR	NR	22% (*n* = 5)	27% (*n* = 6)	0%	4% (*n *= 1)	27% (*n *= 6)	18	NR
2	0%	NR	NR	NR	NR	9% (*n* = 1)	NR	27% (*n* = 3)	NR	NR	NR	Mean: 10.73 Median: 13	Mean: 3.09 Median: 3
3	5.4%	10.9% (*n* = 10)	15.2% (*n* = 14)	8.7% (*n* = 8)	7.6% (*n* = 7)	1.08% (*n *= 1)	9.8% (*n* = 9)	22.8% (*n* = 21)	69.4% (*n* = 25)	NR	3.3% (*n* = 3)	Average: 20.7 (6–89)	Average: 6 (0–74)
4	0%	NR	NR	NR	NR	NR	NR	NR	NR	NR	NR	NR	NR
5	0%	8% (*n* = 3)	5% (*n *= 2)	NR	NR	5% (*n* = 2)	NR	NR	NR	NR	NR	Median: 9 (5–15)	Median: 2 (1–9)
6	1.5% (*n *= 1)	NR	4.4% (*n* = 3)	2.9% (*n *= 2)	NR	2.9% (*n* = 2)	4.4% (*n *= 3)	NR	NR	NR	NR	NR	NR
7	0.6%	NR	NR	9.1% (*n* = 2)	NR	NR	NR	NR	NR	2.3% (*n *= 4)	NR	Median: 5.3 (1–21)	Average: 2.4 (1–14)
8	0%	50% (*n* = 14 recurrence)	14% (*n *= 4)	3% (*n* = 1)	NR	7% (*n* = 2)	NR	NR	NR	NR	NR	NR	NR
9	1% (*n* = 3)	NR	NR	NR	NR	11.3% (*n *= 39)	NR	NR	NR	2.3% (*n* = 8)	NR	NR	NR
10	0%	4% (*n *= 2)	2% (*n *= 1)	NR	NR	NR	12% (*n *= 6)	NR	NR	NR	2% (*n *= 1)	NR	NR
11	1.6% (*n* = 1)	5% (*n* = 3)	NR	NR	NR	3% (*n *= 2)	8% (*n* = 5)	NR	NR	NR	NR	NR	NR
12	0%	12% (*n* = 3)	8% (*n *= 2)	4% (*n *= 1)	NR	NR	NR	4% (*n *= 1)	NR	NR	NR	NR	NR
13	0%	7.7% (*n* = 4)	17.3% (*n* = 9)	NR	NR	NR	1.9% (*n *= 1)	NR	NR	NR	NR	NR	NR
14	0%	8% (*n* = 2)	2% (*n* = 1)	2% (*n* = 1)	NR	2% (*n* = 1)	NR	NR	0%	NR	NR	NR	NR
15	0%	10.8% (*n *= 4)	10.8% (*n* = 4)	NR	NR	10.8% (*n *= 4)	NR	NR	2% (*n* = 1)	5.4% (*n* = 2)	NR	Median: 18 (8–130)	NR
16	0%	NR	9% (*n *= 1)	NR	NR	9% (*n *= 1)	NR	0%	0%	NR	NR	NR	NR

d, days; NR, not reported; LOS, length of hospital stay; ICU, intensive care unit.

## Discussion

A number of prosthetic materials are available for chest wall reconstruction and could be grouped mainly into two categories, rigid and non-rigid materials.

Rigid prostheses, such as methyl methacrylate, silicone, and titanium, have the advantage of ensuring chest wall stability, but on the other hand, rupture, displacement, infection, seroma, and disorders of physiologic respiratory movements were reported (4).

Non-rigid prostheses are meshes and patches, either synthetic or biologic: among synthetic ones are polypropylene, polytetrafluoroethylene, and Vicryl, and among biologic meshes are human bioprosthetic material and bovine pericardium. Advantages of using these consist of the easy manipulation of the material that can be stretched uniformly and sutured easily, providing a scaffold for connective tissue in-growth without significant foreign-body reaction; this is especially true for biologic meshes that allow regeneration stimulating regrowth and revascularization with a lower risk of infection compared to other techniques. Disadvantages are minor protection of intrathoracic organs compared to rigid prostheses since the strength could not be enough to protect them, the cosmetic results, and the higher costs in the case of biologic meshes (21, 4).

Table 3 summarizes rigid and non-rigid prosthetic materials (22, 2, 4).

**Table 3:** A summary of rigid and non-rigid materials used for chest wall reconstruction (22, 4, 2).

	Rigid		Non-rigid
Autologous reconstruction		Bone grafts Vascularized bone	Fascial graft Pedicled muscle Myocutaneous/fasciocutaneous/perforator/omental flaps
Alloplastic reconstruction			
	*Synthetic*	Polymethylmethacrylate Titanium Silicone Ceramic Bone cement	Polyesther (e.g., Mersilene, Dacron, Parietex) Polyglycolic acid (e.g., Dexon) Polylactic acid-expanded polytetrafluoroethylene (e.g., e-PTFE/Gore-Tex) Polydioxanone (e.g., PDS) Polyglactin (e.g., Vicryl) Polypropilene (e.g., Parietene, Marlex, prolene)
	*Bioprosthetic materials*		
	*Allografts*	Human sternal and ribs	Human acellular dermal matrix (e.g., AlloDerm, AlloMax, Flex HD)
	*Xenografts*		Porcine products Small intestine submucosa (e.g., Surgisis) Dermal matrix (e.g., Permacol, CollaMend/XenMtrix, Strattice) Bovine products Pericardium (e.g., Tutopatch, Veritas/Periguard) Dermal matrix (e.g., Surgimend)

According to the results of our review, non-rigid materials for reconstruction of the thoracic wall in tumors have a good safety profile: despite not every study specifically reporting complications such as local infection or necrosis, the mortality rate and the overall risk of complications are always acceptable, also considering the underlying diseases. Furthermore, a common limitation of the reviewed studies is that they rarely report paradoxical respiratory movement, the patient's quality of life, and the patient's satisfaction. We think that these parameters are important outcomes in this kind of surgical procedure since the goals of chest wall reconstruction—apart from the cancer curative intent—are to preserve the cardiac and respiratory function, provide a satisfactory cosmetic result, and, as a direct consequence, improve patients' quality of life.

Interestingly, some studies have compared in a non-randomized way the outcomes of rigid and non-rigid materials. Spicer et al. (13) reviewed 427 patients that underwent chest wall reconstruction, 82 with rigid prostheses and 345 with non-rigid prostheses. No significant difference was found between the two groups in terms of complications and mortality. Complications seem to be related to the extent of resection (number of resected ribs, lobectomy, and pneumonectomy) rather than to the surgical technique used. Similar results were reported by Weyant et al. (23): median length of hospital stay, mortality, and complications did not differ between the rigid and non-rigid prosthesis groups, but a significant clinical difference was found in patients with larger chest wall defects. Kilic et al. (24) compared methylmethacrylate “sandwich” with polytetrafluoroethylene in 59 consecutive patients with chest wall defects larger than 5 cm and located in the anterior part of the thorax: 21 underwent reconstruction with polytetrafluoroethylene and 38 with methylmethacrylate “sandwich.” No significant differences were found in mortality, but paradoxical respiratory movement was higher in the polytetrafluoroethylene group as well as the mean length of hospital stay was longer.

A comparison between different non-rigid materials was performed in a few studies. Huang et al. (19) retrospectively compared patients that underwent chest wall reconstruction with Gore-Tex (*n *= 18), with autologous flaps (*n *= 14), or other meshes (Marlex, Medifit) (*n *= 5). They found a higher chest drainage time in the Gore-Tex group and one empyema in the Marlex group, and no differences were found in the shrinkage of the materials among the three groups. Pneumonia was described in two patients treated with Gore-Tex and in one patient with autologous flap and mesh. Regarding paradoxical movement, one case in the Gore-Tex group was reported. Two cases of infections were found in the flap and in the Gore-Tex group.

Other similar pieces of evidence come out from the study by Hanna et al. (9): postoperative outcomes of non-rigid meshes and autologous flaps were similar between those with a chest wall defect <60 cm^2^ and those with a defect >60 cm^2^; in the subgroup analysis of the large-defect cohort, the incidence of admission in intensive care unit was higher in the mesh group; conversely the rate of reoperation and local infection was higher in the mesh group, but none of the above-mentioned parameteres reached statistical significance.

The study from Girotti and colleagues (17) described specifically the use of semi-rigid material after sternectomy: the mortality was 0%, but the rate of infection was higher reported among the selected studies. It was not specified which type of rigid material was used, although data from the literature suggested that Gore-Tex dual mesh performs well in sternal reconstruction (20).

Thus, given the paucity and the design of the studies on the topic, no strong conclusions could be drawn about the best material to be used.

Moreover, it has to be underlined that in the majority of reports a combination of non-rigid and rigid prostheses is reported, with the rationale of exploiting the different features of materials, especially in the case of reconstruction of large chest wall defects. One of the most common composites is the combination of polymethylmethacrylate in two layers of the propylene mesh, placed as a “sandwich” (21), but several materials could be mixed, such as rigid+non-rigid synthetic prostheses, autologous + synthetic prostheses, xenograft + synthetic prostheses, and so on. The report by Heo et al. (25) is an example of how rigid and non-rigid synthetic prosthetic meshes along with xenografts could be combined at the same time: in six patients, after the removal of the chest wall tumor, the visceral pleura was repaired with Gore-Tex; the first layer of the acellular dermal matrix was placed on Gore-Tex, then bone cement was used as a substitute for ribs, and finally the second layer of the acellular dermal matrix was sutured with the first one, preventing the contact of the cement with the soft tissue. No major complications occurred, and no perioperative mortality was reported.

This strengthens what was stated before about the choice of the prosthetic material, which has to be related to the patient's chest wall defect in terms of the dimension and location in the thorax and to the experience and preference of the operator, rather than on a proven superiority of a technique.

Finally, soft tissue replacement is part of the procedure of chest wall reconstruction, but it is seldom used alone as a technique of reconstruction because of the lack of rigidity, especially in large defects: it consists of transporting/transplanting muscles or part of the greater omentum along with the skin for the closure of thoracic defects. Muscles commonly used are the pectoralis major, latissimus dorsi, and rectus abdominis muscles. Also, omentoplasty has been used due to its good vascularization pattern and immunological property; it also requires a skin graft as coverage (26).

## Conclusions

In conclusion, to date, no “gold standard” procedure is recommended for chest wall reconstruction. The surgical procedure should be tailored to the patient's clinical status, the underlying disease, and the dimension and the location of the chest wall defect, as well the use of prosthetic materials should be decided on the basis of local availability, cost, and, more importantly, the surgical experience of the operator. Non-rigid prostheses, used alone or in combination with other materials, offer good outcomes from either a clinical or a safety point of view. Further studies are necessary to investigate the patients' quality of life and patient's satisfaction with aesthetic results.

## Data Availability

The raw data supporting the conclusions of this article will be made available by the authors without undue reservation.
