# Supplementary material for: Chest wall reconstruction in benign and malignant tumors with non-rigid materials: An overview
Source: Front Surg. 2022 Aug 3;9:976463. doi: 10.3389/fsurg.2022.976463 (PMC9381953; doi:10.3389/fsurg.2022.976463)
Supplement: Supplementary file 1 [file Data_Sheet_4_v1.pdf]

July 09, 2022

Editor in Chief
of Frontiers in Surgery

Dear Editor,

I would like to re-submit to your attention our manuscript: "**Chest wall reconstruction in benign and malignant tumors with non-rigid materials: an overview**", to be published in the Frontiers in Surgery, in the category "Review".

This article is invited by Drs. Alessandro Gonfiotti, Roberto Crisci, Laura Socci, Antonino Morabito for the collection *Chest Wall Surgery in Adults and Pediatrics*.

I would like to thanks the reviewers. Following the considerations of the reviewer 3, We changed the tile of manuscript.

The material has not been previously published or submitted elsewhere for publication and will not be sent to another journal until a decision is made concerning publication by **Frontiers in Surgery.**

The study is not funded by any Industry.

The authors have no conflicts of interest to declare.

The authors are accountable for all aspects of the work in ensuring that questions related to the accuracy or integrity of any part of the work are appropriately investigated and resolved.

All tables are original.

I am looking forward to hearing from you soon.

With my best regards.

Duilio Divisi
